# A homodimeric complex of HLA-G on normal trophoblast cells modulates antigen-presenting cells *via* LILRB1

**DOI:** 10.1002/eji.200737089

**Published:** 2007-07

**Authors:** Richard Apps, Lucy Gardner, Andrew M Sharkey, Nick Holmes, Ashley Moffett

**Affiliations:** Department of Pathology, Cambridge UniversityCambridge, UK

**Keywords:** Allogeneic fetal survival, Decidual leukocytes, HLA-G, Human trophoblast, Leukocyte immunoglobulin-like receptors

## Abstract

In healthy individuals, the non-classical MHC molecule HLA-G is only expressed on fetal trophoblast cells that invade the decidua during placentation. We show that a significant proportion of HLA-G at the surface of normal human trophoblast cells is present as a disulphide-linked homodimer of the conventional β_2_m-associated HLA-I complex. HLA-G is a ligand for leukocyte immunoglobulin-like receptors (LILR), which bind much more efficiently to dimeric HLA-G than to conventional HLA-I molecules. We find that a LILRB1-Fc fusion protein preferentially binds the dimeric form of HLA-G on trophoblast cells. We detect LILRB1 expression on decidual myelomonocytic cells; therefore, trophoblast HLA-G may modulate the function of these cells. Co-culture with HLA-G^+^ cells does not inhibit monocyte-derived dendritic cell up-regulation of HLA-DR and costimulatory molecules on maturation, but did increase production of IL-6 and IL-10. Furthermore, proliferation of allogeneic lymphocytes was inhibited by HLA-G binding to LILRB1/2 on responding antigen-presenting cells (APC). As HLA-G is the only HLA-I molecule that forms β_2_m-associated dimers with increased avidity for LILRB1, this interaction could represent a placental-specific signal to decidual APC. We suggest that the placenta is modulating maternal immune responses locally in the uterus through HLA-G, a trophoblast-specific, monomorphic signal present in almost every pregnancy.

See accompanying commentary: http://dx.doi.org/10.1002/eji.200737515

## Introduction

HLA-G is a non-classical HLA class I (HLA-I) molecule, selectively expressed in healthy individuals by fetal placental trophoblast cells that invade maternal uterine tissues during placentation 1–4. HLA-G is encoded in the MHC on chromosome 6 and displays a typical HLA-I structure, including association with β_2_m and peptide. Unusual features of HLA-G include absence of an endosomal recycling motif, leading to a prolonged cell surface half life 5. Minimal polymorphism 6, 7, an endoplasmic reticulum retrieval mechanism 8, and the structure of the peptide-binding groove 9 contribute to a restricted peptide repertoire *in vivo* 10. These characteristics argue against a role for HLA-G as a conventional HLA-I molecule presenting pathogen-derived peptides to T cells. The functions of HLA-G are not clear, although restricted expression to extravillous trophoblast (EVT) cells at the maternal-fetal interface suggests a role in normal pregnancy. Fetal EVT cells invading the decidua and myometrium come into close contact with maternal uterine leukocytes that, in early gestation, are predominantly composed of NK cells, as well as myelomonocytic cells and some T cells 11.

Receptors for HLA-G have been described on NK and myelomonocytic cells derived from peripheral blood. The NK cell receptor is KIR2DL4, a member of the killer immunoglobulin-like receptor (KIR) family. NK cells are reported to endocytose HLA-G into KIR2DL4-containing compartments, and the subsequent interactions modulate NK cell cytokine secretion 12. LILRB1 and LILRB2 are inhibitory receptors of the leukocyte immunoglobulin-like receptor (LILR) family which are mainly expressed by DC and macrophages 13. LILRB1 and B2 bind most HLA-I molecules 13–15, but the highest affinity is for HLA-G 16.

Recently, it was discovered that HLA-G can exist as a dimer in addition to the conventional heterotrimeric HLA-I molecule conformation. Dimers of HLA-G were first described with recombinant protein *in vitro* 17, then later observed on the surface of both transfected cells 17, 18 and the choriocarcinoma cell line JEG-3 19. The HLA-G dimer is linked by disulphide-bonding between cysteine 42 residues of the heavy chain α1 domain, an exposed extracellular cysteine not involved in intramolecular Ig superfamily domain formation 18, 20. Comparison with other HLA-I gene sequences shows that a cysteine residue at this position is unique to HLA-G 18. Amongst other primates, cysteine 42 is conserved in the MHC-G genes of chimpanzees and gorillas that are also non-polymorphic and presumably act as functional homologues. However, in the orang-utans, Old and New World monkeys where MHC-G orthologues function as classical MHC-I molecules or are pseudogenes 21, cysteine 42 is substituted to a serine which has been shown to abrogate dimerisation 9, 18.

The outstanding questions are whether these HLA-G dimers are formed *in vivo* on normal trophoblast cells and what their function might be at the maternal-fetal interface. We now show that a significant proportion of the HLA-G present on the surface of normal first-trimester trophoblast cells exists as a dimer. This complex is a homodimer of two conventionally β_2_m-associated HLA-G molecules. We demonstrate LILRB1 and B2 expression by decidual myelomonocytic cells and show that a LILRB1-Fc fusion protein preferentially binds to the dimeric form of HLA-G on normal trophoblast cells. Therefore, HLA-G dimers can provide a trophoblast-specific signal to decidual myelomonocytic leukocytes *via* LILRB1. We present evidence that this interaction leads to modulation of dendritic cell (DC) function and suppression of allogeneic lymphocyte proliferation. In this way, HLA-G potentially modifies the maternal local immune response to fetal trophoblast cells.

## Results

### HLA-G forms a disulphide-linked homodimer of the conventional, β_2_m-associated HLA class I complex

**Figure 1:**
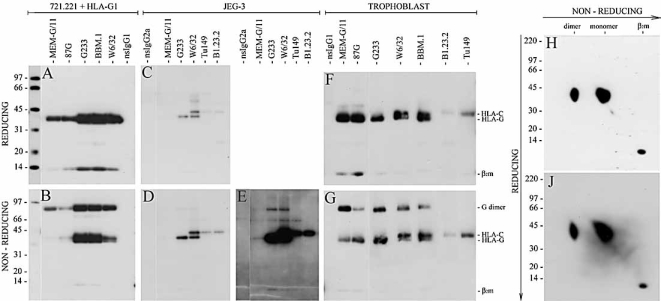
HLA-G is present on the surface of normal trophoblast cells as a disulphide-linked homodimer of the conventional β_2_m-associated class I complex. 721.221 + HLA-G1 transfectants (A, B), JEG-3 cell line (C–E) and trophoblast (F, G) were surface biotinylated, immunoprecipitated with the indicated antibodies and resolved by reducing (A, C, F) or non-reducing (B, D, E, G) PAGE before Western blotting with streptavidin-HRP. All were exposed for less than 1 min, with the exception of (E) which was exposed for 10 min. Complexes immunoprecipitated with G233 from 721.221 HLA-G^+^ transfectants (H) and trophoblast (J) were also analysed by 2D-PAGE. These gels are representative of at least three different experiments for each cell type, using a total of 20 pooled samples for trophoblast cells. Images in this figure include lanes from several gels.

To detect HLA-G dimers, surface proteins of 721.221 cells transfected with HLA-G1 cDNA were biotinylated and immunoprecipitated with a panel of HLA-I-reactive antibodies. HLA-G-specific MEM-G/11, 87G and G233 recognise the β_2_m-associated class I structure. W6/32 is also conformationally dependant but binds all HLA-I molecules. The immunoprecipitated complexes were first analysed by denaturing reducing PAGE, followed by Western blotting for biotin-labelled surface proteins. As expected, both HLA-G-specific and pan-class I antibodies detected an HLA-G band at 39 kDa, as well as co-immunoprecipitating the 13.7-kDa β_2_m molecule (Fig. 1A). However, when analysed under denaturing but non-reducing conditions, an additional 80-kDa band appears, indicating that some of the HLA-G is present as a disulphide-linked homodimer (Fig. 1B). Densitometry measurements of complexes detected by G233, MEM-G/11, W6/32 and BBM.1 estimate that 40% of the HLA-G molecules are in the dimeric form. HLA-G dimerisation may alter or obscure the 87G epitope because this antibody preferentially binds the monomeric form of HLA-G compared to other antibodies in the panel. Under non-reducing conditions, both 39- and 80-kDa bands can be co-immunoprecipitated by the anti-β_2_m antibody, BBM.1, indicating that the HLA-G dimers are β_2_m associated (Fig. 1B). Therefore, HLA-G dimers are of the conventional class I complex and not free heavy chains as described previously for HLA-B27 22. All these experiments were repeated with 721.221 cells transfected with genomic HLA-G (as opposed to cDNA) and the rodent cell line, Ltk, transfected with human β_2_m and HLA-G with similar results (not shown).

The characteristics of HLA-G, when expressed by a trophoblast cell line, were investigated using the choriocarcinoma cell line, JEG-3. This is similar to normal EVT in that it expresses HLA-C, HLA-G and HLA-E whilst lacking HLA-A and HLA-B 10, 23, 24. Under reducing conditions, an HLA-G band at 39 kDa, with an additional 45-kDa molecule, was detected by W6/32 (Fig. 1C). The 45-kDa species was confirmed as a classical HLA-I molecule by immunoprecipitation with Tü149 and B1.23.2, antibodies specific for HLA-C on these cells (Fig. 1D, E). In contrast to the findings with HLA-G transfectants, no 80-kDa band was observed under non-reducing conditions unless the blot was overexposed to the point that significant background staining was apparent (Fig. 1D, E). Densitometry measurements suggest that less than 10% of the HLA-G molecules are dimerised. Thus, the conventional monomeric form of HLA-G predominates on JEG-3 cells, a finding consistent with previous reports 17, 19.

### The dimeric HLA-G complex is expressed at the surface of normal trophoblast cells

The outstanding question is whether the dimeric form of HLA-G is present on the surface of normal trophoblast cells. Cells isolated from normal first-trimester placentae, in which only trophoblast cells have been shown to express HLA-G 25, were surface biotinylated and immunoprecipitated as before. Immunoprecipitation with HLA-G-specific antibodies detected 80-kDa dimeric HLA-G as well as the conventional 39-kDa form under non-reducing conditions (Fig. 1G). In contrast, the 80-kDa species was not seen in reducing PAGE (Fig. 1F). By densitometry, 40% of the HLA-G molecules appear to be in the dimeric complex, although the mAb 87G displays a greater bias to the monomer as before. W6/32 and BBM.1 antibodies immunoprecipitated both the monomeric and dimeric forms of HLA-G, and the 45-kDa HLA-C molecule was also expressed by normal trophoblast cells 23, 24. As with JEG-3, HLA-C-specific antibodies confirm the identity of the bands in this doublet (Fig. 1F, G).

To confirm that the 80-kDa complex consists of homodimers of HLA-G molecules and not of heterodimers of different HLA-I molecules, or is a cross-reaction of the Western reagents with an unrelated 80-kDa protein, two-dimensional (2D)-PAGE was performed. Surface-labelled HLA-G was specifically immunoprecipitated from transfectants (Fig. 1H) and trophoblast (Fig. 1J) with the mAb G233. Non-reducing PAGE in the horizontal dimension resolved the 80-kDa complex, 39-kDa conventional HLA-G and 13.7-kDa β_2_m. After reduction on the gel followed by reducing PAGE in the vertical dimension, the previously 80-kDa species migrated the same distance as conventional 39-kDa HLA-G. This confirms that the 80-kDa species is a disulphide-linked homodimer of the 39-kDa HLA-G molecule.

To summarise, these results show that HLA-G can form disulphide-linked homodimers that are β_2_m associated and that a significant proportion of the HLA-G molecules at the surface of normal trophoblast cells *in vivo* are present in this dimeric conformation. Similar results were obtained for HLA-G expressed by transfected 721.221 and Ltk cells. Preferential binding of 87G to the monomer in both transfectants and trophoblast cells suggests that the dimer has a similar conformation in both contexts, and therefore these transfectants represent a good model of HLA-G as displayed at the surface of the trophoblast. In contrast, HLA-G is found on the surface of the cell line, JEG-3, predominantly as the conventional monomeric class I complex.

### Soluble HLA-G also forms a disulphide-linked homodimer

**Figure 2:**
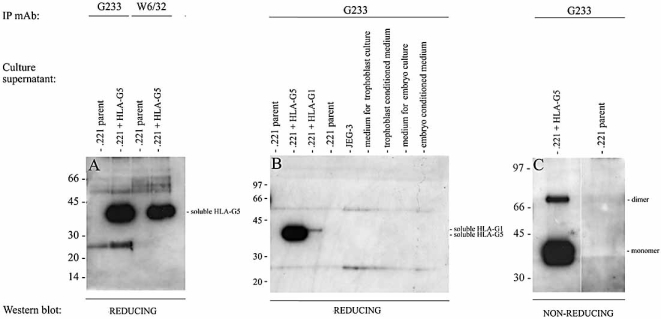
Soluble HLA-G can also exist as a disulphide-linked homodimer of the conventional β_2_m-associated class I complex. Soluble HLA-G molecules were immunoprecipitated with the indicated antibodies from supernatants of 721.221 cells transfected with HLA-G1 or HLA-G5 cDNA compared to untransfected cells. The JEG-3 cell line, isolated primary trophoblast, or pre-implantation embryo culture supernatants were also immunoprecipitated compared to unconditioned medium. Precipitated complexes were separated by reducing (A, B) or non-reducing PAGE (C) and detected by Western blotting with biotinylated mAb MEM-G/1 followed by streptavidin-HRP. HLA-G1^+^ transfectant, JEG-3 and trophoblast culture supernatants were concentrated 30 times before immunoprecipitation. Gels are representative of four independent experiments. The image in (C) includes lanes from separate gels.

We also investigated whether the two soluble forms of HLA-G form dimers. Full-length membrane-bound HLA-G1 molecules can be shed intact or cleaved from the surface by metalloproteinase activity 26, 27. Also, a splice variant, HLA-G5, retains intron 4, introducing a premature stop codon before the transmembrane domain 28. Both of these soluble forms of HLA-G were investigated by immunoprecipitation with mAb G233 and W6/32 from culture supernatant of 721.221 cells transfected with either HLA-G1 or HLA-G5 cDNA. Immunoprecipitated complexes were analysed by reducing or non-reducing PAGE and detected on a Western blot with the HLA-G-specific mAb MEM-G/1. Under reducing conditions, secreted HLA-G5 was detected at ∼37 kDa, as expected (Fig. 2A). A soluble HLA-G band was immunoprecipitated from supernatants of HLA-G1-transfected cells, but this was at much lower concentration and only detected in 30-fold concentrated samples (Fig. 2B). We looked for the presence of dimers using non-reducing conditions and found that a small proportion of HLA-G5 was present as a dimer, using both G233 (Fig. 2C) and W6/32 mAb (not shown). This confirms that soluble HLA-G can form a disulphide-linked homodimer of the β_2_m-associated class I complex similarly to the transmembrane molecule. Immunoprecipitations were repeated using concentrated supernatant of cultured primary trophoblast and pre-implantation embryos, but no soluble HLA-G1 or HLA-G5 was detected (Fig. 2B). In conclusion, the HLA-G5 soluble splice variant of HLA-G can exist as a dimer identically to HLA-G1 at the cell surface. However, no soluble forms of HLA-G were detected *in vivo*.

Artefactual multimerisation can occur due to incomplete denaturation under non-reducing conditions permitting hydrophobic associations. However, cysteine 42 substitution 18 and the dimer crystal structure 20 have confirmed that HLA-G homodimers are disulphide linked. Cytoplasmic tail cysteine residues have also been observed to mediate post-lysis dimerisation of classical HLA-I molecules when released from the reducing intracellular environment 29. To avoid post-lysis disulphide bond formation, in our experiments a lysis buffer containing the alkylating agent iodoacetamide was used that prevents *de novo* disulphide bond formation between HLA-I molecules 30. In addition, the HLA-G sequence lacks cytoplasmic cysteine residues and we have also shown that the soluble HLA-G5 splice variant product forms dimers (Fig. 2). We note that the β_2_m band was consistently stronger in reducing blots. This is likely to be an artefact due to residual tertiary conformation under non-reducing conditions stearically hindering access of streptavidin-HRP to biotinylated residues. Reprobing of blots with anti-β_2_m mAb confirmed that all light chain was at 13.7 kDa (not shown) and weaker bands from some biotinylated molecular weight markers also occurred under non-reducing conditions (Fig. 1). We did not investigate free heavy chains of HLA-G, which have been observed at the surface of transfectants 19, due to the lack of an antibody recognising free heavy chains that is specific for HLA-G *in vivo* 31.

### LILRB1, an HLA class I molecule receptor, is expressed by decidual leukocytes

**Figure 3:**
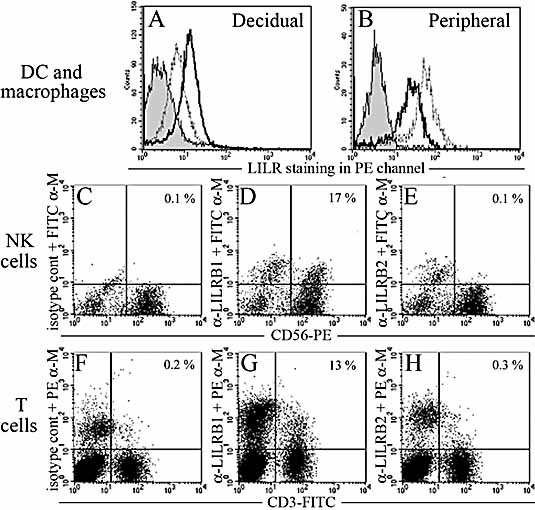
LILRB expression by decidual leukocytes. Decidual (A) or peripheral (B) leukocytes were identified as myelomonocytic cells by forward/side scatter and HLA-DR^+^ gates and stained with anti-LILRB1 (dark line), anti-LILRB2 (grey line) or isotype control antibodies (filled histogram). Decidual CD56^+^ (C–E) or CD3^+^ (F–H) lymphocytes were stained with isotype control (C, F), anti-LILRB1 (D, G) and anti-LILRB2 (E, H) antibodies. These results from decidual leukocytes are representative of seven different experiments using decidua pooled from 17 samples. Similar results for both LILRB1 and 2 were observed with two different antibodies. The apparently higher isotype control mAb staining of T cells compared to NK cells results from inclusion of more cells in plots demonstrating T cell staining, due to their low frequency in decidual leukocyte populations.

HLA-G binds to members of the LILR family, LILRB1 and B2 13, 14. Although these receptors bind a broad spectrum of HLA-I alleles, they have a higher affinity for HLA-G 16, particularly in its dimeric form 18, 20. Therefore, we investigated LILRB1 and B2 expression by decidual leukocytes. Two different antibodies were used for both receptors. Maternal decidual leukocytes were isolated from first-trimester pregnancies and identified as T cells (CD3), NK cells (CD56) and DC or macrophages (HLA-DR) 32, followed by staining for LILR expression. LILRB1 was detected on all DC and macrophages (Fig. 3A), as well as 20% of NK cells (Fig. 3D) and 10% of T cells (Fig. 3G). Expression of LILRB2 was restricted to DC and macrophages (Fig. 3A, E, H). LILRB2 is expressed more strongly on peripheral than decidual myelomonocytic cells, whereas LILRB1 appears to predominate in the decidua (Fig. 3A, B).

### LILRB1-Fc fusion protein preferentially binds the dimeric form of HLA-G *in vivo*

**Figure 4:**
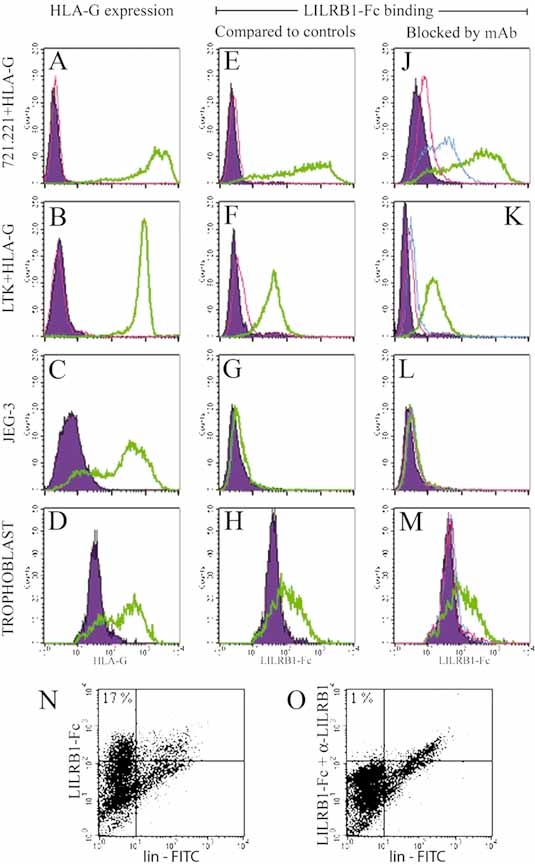
LILRB1-Fc binds to HLA-G at the surface of transfected cells and trophoblast. HLA-G expression on the indicated cells was detected by G233 staining (green) compared to untransfected cells (red) or with an isotype control mAb (filled histogram) (A–D). LILRB1 binding to the indicated cells was detected by LILRB1-Fc staining (green) compared to untransfected cells (red) or with an IgG-Fc fragment (filled histogram) (E–H). Specificity was confirmed with blocking mAb (J–M): LILRB1-Fc (green) and IgG1-Fc fragment (filled histogram) binding are shown as before and also after pre-incubation of the fusion protein with anti-LILRB1 mAb M401 (red) or of the cells with anti-HLA-I mAb W6/32 (blue). These results are representative of four different experiments for each cell type, using trophoblast from eight samples. To see LILRB1-Fc binding to trophoblast cells, contaminating leukocytes that bound the fusion protein non-specifically *via* FcγR were labelled with a lineage marker cocktail (CD3/14/16/19/20/56-FITC) and analysis was performed on FITC-negative trophoblast cells (upper left quadrant). Dot plots of LILRB1-Fc binding (N) and blocking by anti-LILRB1 mAb (O) are shown.

Predominant expression of LILRB1 by decidual leukocytes suggests that this receptor may be particularly important in the recognition of trophoblast HLA-I molecules. To investigate this possibility, we studied binding of a LILRB1-Fc fusion protein to cells expressing HLA-G. As predicted, LILRB1-Fc bound to 721.221 and Ltk cells transfected with HLA-G (Fig. 4E, F). The level of binding correlated with HLA-G expression (Fig. 4A, B) and the specificity was demonstrated by blocking with mAb to LILRB1 (M401) and W6/32 which is known to block LILRB1 binding to HLA-I molecules 33 (Fig. 4J, K). HLA-G-specific mAb do not bind epitopes blocking LILRB1 binding. To detect LILRB1-Fc binding to primary trophoblast preparations, it was necessary to first label contaminating leukocytes expressing FcγR with lin-FITC, leaving trophoblast and stromal cells in the FITC-negative quadrants (Fig. 4N, O). A gate around these cells shows that the level of HLA-G was less abundant at the surface of normal trophoblast than transfectants (Fig. 4A, B, D). Despite this, binding of the LILRB1-Fc fusion protein to trophoblast cells could still be demonstrated and was blocked by mAb to LILRB1 or HLA-I (Fig. 4H, M, N, O).

Interestingly, no binding of LILRB1-Fc to JEG-3 cells was detected (Fig. 4G, L) even though these cells showed higher levels of HLA-G expression than trophoblast cells (Fig. 4C, D). The failure to detect binding to JEG-3 cells, which display HLA-C, HLA-E and mainly monomeric HLA-G, suggests that LILRB1-Fc preferentially binds the dimeric form of HLA-G and not conventional HLA-I molecules.

**Figure 5:**
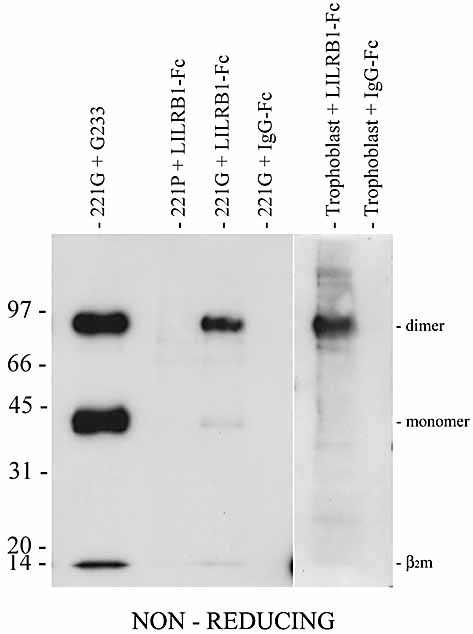
LILRB1-Fc preferentially binds to the dimeric form of HLA-G at the cell surface. 721.221 HLA-G^+^ transfectants (221G), untransfected controls (221P) and normal trophoblast cells were surface-biotinylated, immunoprecipitated with the indicated antibodies or fusion proteins and resolved by non-reducing PAGE before Western blotting with streptavidin-HRP. These results are representative of five different experiments for each cell type. Trophoblast cells were pooled from 15 samples. The image includes lanes from separate gels.

To test this, the LILRB1-Fc fusion protein was used to immunoprecipitate surface-labelled proteins from HLA-G-transfected and primary trophoblast cells (Fig. 5). As before, the G233 mAb detects both monomeric and dimeric HLA-G, with ∼40% of HLA-G molecules in the dimeric form. The LILRB1-Fc fusion protein strongly bound the dimeric form of HLA-G, monomeric HLA-G was only very weakly detected, and the 45-kDa classical HLA-C molecule expressed by trophoblast cells was not seen at all. These bands were confirmed to be specific for an interaction of LILRB1 with HLA-G because they were not precipitated from untransfected 721.221 cells or by an IgG1-Fc fragment. That LILRB1-Fc predominantly binds to dimeric rather than monomeric HLA-G at the surface of HLA-G transfectants and normal trophoblast cells suggests that this is likely to be the major interaction occurring *in vivo*.

### Monocyte-derived DC activated in the presence of HLA-G demonstrate altered cytokine production

**Figure 6:**
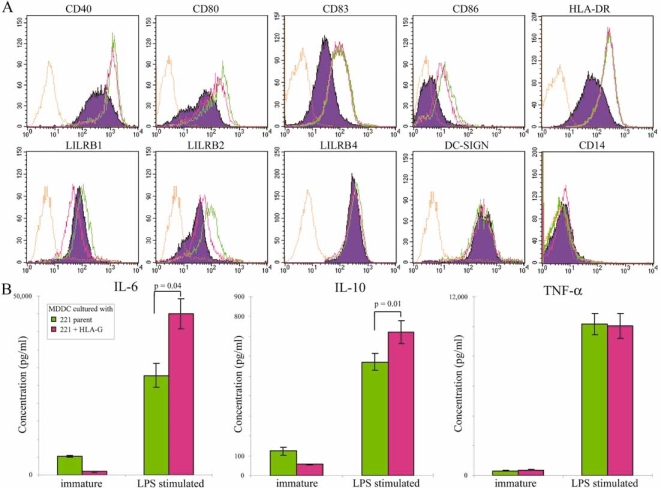
MDDC activated in the presence of HLA-G show normal up-regulation of maturation markers but altered cytokine production. (A) MDDC, co-cultured with HLA-I null 721.221 cells, were analysed for expression of the indicated LILR receptors, differentiation and activation markers in the absence of stimulation (filled histogram) or after exposure to LPS (green trace). The same markers were analysed after exposure to LPS in the presence of HLA-G transfectants instead of HLA null cells (red trace). Isotype control staining for each marker is shown in orange. (B) Levels of the indicated cytokines in these co-culture supernatants were measured by ELISA on two separate occasions. A representative experiment is shown here, ± SD of triplicate measurements. Culture supernatants from at least four independent experiments were tested and analysed together for statistical significance.

Given the high-affinity interaction of HLA-G dimers with LILRB1 and the expression of this receptor by all decidual HLA-DR^+^ cells, we next investigated whether HLA-G appeared to have any functional effect on DC. Due to the difficulty in obtaining sufficient HLA-DR^+^ cells from the decidua that are not activated by the isolation procedure 32, we used monocyte-derived DC (MDDC) generated *in vitro*. MDDC were prepared from peripheral blood, and their response to a maturation stimulus was observed in co-culture with cells expressing HLA-G. We used 721.221 or Ltk cells displaying dimeric and monomeric HLA-G identical to normal trophoblast and untransfected cells as controls. MDDC maturation was stimulated by TLR4 ligand (LPS), inflammatory cytokines (TNF-α, IL-6 and IL-1β) or soluble CD40 ligand. During MDDC differentiation, HLA-G was presented either concomitantly with the maturation stimulus or preceding it. The results were essentially similar. MDDC expressed the LILRB1 and B2 receptors, and these were down-regulated in co-culture with cells expressing HLA-G but not with control cells (Fig. 6A). This is consistent with an interaction occurring between the LILR and HLA-G. HLA-DR, costimulatory molecules (CD40, CD80, CD86) and the activation marker CD83 were up-regulated on MDDC in response to all three maturation stimuli (Fig. 6A). Up-regulation of these markers was not affected by HLA-G and is consistent with maturation to an antigen-presenting phenotype. However, analysis of the cytokines produced by these activated MDDC did show changes when HLA-G was present. MDDC activated in the presence of HLA-G demonstrated a significant increase in IL-6 and IL-10 production (*p* = 0.04 and *p* = 0.01, respectively) (Fig. 6B). In contrast, there was no effect on TNF-α (Fig. 6B) or IL-8 secretion (not shown). These experiments indicate that when DC are activated, although HLA-G does not affect the acquisition of maturation markers, it does alter their cytokine secretion by increasing IL-6 and IL-10 production.

### HLA-G stimulating LILRB on HLA-DR^+^ cells results in inhibited allogeneic lymphocyte proliferation

We next investigated how DC exposed to HLA-G would function in the presentation of allogeneic stimuli to T cells. Previously, the presence of HLA-G in an MLR has been shown to result in reduced responder T cell proliferation 34–36. Most reports infer that HLA-G is directly inhibiting T cells, although a molecular mechanism has not been defined as HLA-G-restricted TCR have not been described in humans 34, 35. Given the strong binding of dimeric HLA-G to LILRB1 and the observed increased IL-6 and IL-10 production by MDDC exposed to HLA-G, we sought to determine whether it is the interaction between trophoblast HLA-G and LILRB on antigen-presenting cells (APC) that results in down-regulation of allogeneic T cell proliferation.

**Figure 7:**
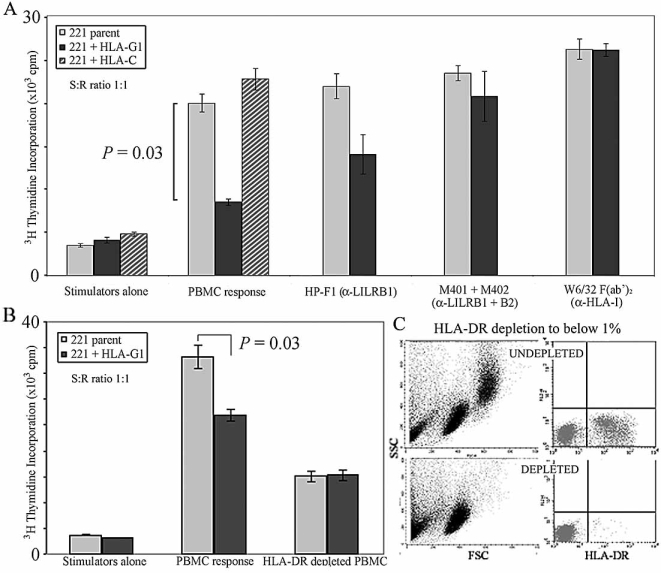
Allogeneic lymphocyte proliferation is inhibited by HLA-G interacting with LILRB receptors on HLA-DR^+^ cells. (A) Irradiated 721.221 stimulator cells transfected with various HLA-I molecules were cultured with PBMC responders from a healthy donor. Proliferation measured by [^3^H]thymidine incorporation after 5 days was reduced in the presence of HLA-G but not a classical HLA-I molecule. Reduced proliferation to HLA-G transfectants was abrogated by addition of anti-LILRB and anti-HLA-I mAb. (B) When HLA-DR^+^ cells were depleted from the responder population, a reduced proliferative response with no additional inhibitory effect by HLA-G was observed. One result is shown, ± SD of triplicates. Three to ten independent experiments under each condition were performed and these were analysed together for statistical significance. (C) Flow cytometry analysis of the responder population confirming depletion of HLA-DR^+^ cells.

We therefore established a one-way MLR using PBMC as responders and 721.221 cells as stimulators. The stimulators were either HLA-I null (parent), HLA-G1 transfectants expressing HLA-G dimers as well as monomers, or HLA-C transfectants to control for the effect of a conventional HLA-I molecule. At a 1 : 1 stimulator/responder ratio, the presence of HLA-G reduced the alloproliferative response compared to the parent cell line, by a magnitude consistent with previous reports (*p* = 0.03) (Fig. 7A) 34–36. In contrast, a marginally increased response was seen with HLA-C transfectants (Fig. 7A) 34. Consistent with previous results 36, mAb specific to HLA-I or LILRB1/2 molecules that are known to block HLA-G:LILRB binding (Fig. 4) 33, 37 reversed the inhibition of proliferation observed in the presence of HLA-G (Fig. 7A).

To test whether HLA-DR^+^ cells expressing LILRB were responsible for the HLA-G-mediated inhibition of proliferation, we next depleted these cells from responder populations. In an MLR, responder T cell proliferation results both from direct stimulation by the allogeneic HLA of stimulator cells as well as from indirect stimulation of allogeneic stimulator cell antigens presented by responder APC. We found that if HLA-DR^+^ cells are depleted from the responding PBMC population (Fig. 7C), the proliferative response to the parent cell line is reduced as expected, since only directly reacting T cells can respond (Fig. 7B). However, there is no additional inhibitory effect in the presence of HLA-G after removal of HLA-DR^+^ cells from the responding PBMC. Therefore, we conclude that HLA-G only inhibits the indirect APC-dependant stimulation of proliferation. Taken together, our findings indicate that the interaction of HLA-G dimers with LILR expressed by HLA-DR^+^ cells is directing myelomonocytic cells to down-regulate T cell proliferative responses.

## Discussion

We have shown that a significant proportion of HLA-G molecules present at the surface of normal human trophoblast cells *in vivo* exist as a dimer. Previously, there were concerns that these dimers were merely an artefact of overexpression in transfectants, particularly as we and others have found that they are difficult to detect on JEG-3 choriocarcinoma cells 17, 19. This homodimer is of the conventional, β_2_m-associated HLA-I complex. Crystal structure 20 and mutational studies 18 have demonstrated that the dimeric form of HLA-G is formed by disulphide bonding between cysteine 42 residues of the heavy chain α1 domain. This β_2_m-associated dimeric complex is likely to be unique to HLA-G, as among HLA-I molecules a cysteine at position 42 is specific to HLA-G. HLA-B27, for example, can also form disulphide-linked multimers *in vitro*, but these are mediated by bonding between cysteines at position 67 and require heavy chain dissociated from β_2_m 22. The unique conformation of HLA-G as a homodimer of conventional, β_2_m-associated class I complexes crucially maintains recognition by the LILRB1 receptor because co-crystal structures confirm that a significant proportion of this receptor's interface with HLA-I molecules is with the β_2_m molecule 15. In healthy individuals, HLA-G expression is restricted to fetal trophoblast cells that invade the uterine decidua and myometrium in placentation 11. It is possible that HLA-G dimers could act as a pregnancy-specific signal modulating the local uterine immune response.

The HLA-G dimers we have demonstrated at the surface of EVT will come into contact with virtually all the maternal leukocytes of the decidua 11. The dominant population of NK cells may bind endocytosed HLA-G *via* KIR2DL4 12, although it is not known if HLA-G dimerisation affects KIR2DL4 binding. In contrast, preferential binding of the HLA-G dimers to LILR molecules has been well described. The inhibitory receptors, LILRB1 and LILRB2, both bind all HLA-I molecules, with a higher affinity for HLA-G attributed to sequence differences in the α3 domain 16. HLA-G free heavy chains observed at the surface of transfectants reduce LILRB1 binding to β_2_m-associated HLA-G complexes and potentially modulate LILRB1 stimulation 19. Recently, the increased avidity of HLA-G dimerisation has been shown to substantially increase binding to LILRB. A LILRB1-Fc fusion protein bound more strongly to wild-type HLA-G than to HLA-G where the cysteine is mutated to a serine at position 42, abrogating dimerisation 18, 19. This was supported by surface plasmon resonance measurements of recombinant monomeric and dimeric HLA-G complexes binding LILRB molecules 20. The increased binding avidity of HLA-G dimers translates into augmented signalling through LILRB1. This was shown by LILRB1-mediated inhibition of serotonin release triggered by IgεR, inhibition of NK killing 18 and a LILRB1 chimera NFAT-GFP reporter cell assay 20.

We therefore investigated LILR expression in the decidua and binding to HLA-G dimers on trophoblast cells. Previous studies in humans have only demonstrated binding of HLA-G tetramers to peripheral myelomonocytic cells 38. We detected LILRB1 and B2 expression by all decidual HLA-DR^+^ cells, consistent with results detecting expression on decidual macrophages 39. In contrast to peripheral myelomonocytic cells, LILRB1 and not LILRB2 appears to be predominantly expressed in the decidua. LILRB1 was also detected on 20% of NK and 10% of T cells in the decidua. This is similar to the expression on peripheral PBMC 13, 40, although the function of LILRB1 on these other leukocytes remains unclear.

Having detected LILRB expression by decidual myelomonocytic cells, we demonstrated binding of LILRB1-Fc fusion proteins to HLA-G at the surface of normal trophoblast cells. The specificity of this interaction was confirmed by blocking mAb to both LILRB1 and HLA-I molecules. In keeping with the previous findings that LILRB1 preferentially binds to HLA-G dimers, no binding was demonstrable with JEG-3 cells that mainly displayed conventional monomeric HLA-G. A more direct demonstration that LILRB1 interacts preferentially with HLA-G dimers on trophoblast cells was shown by the LILRB1-Fc fusion protein predominantly immunoprecipitating dimeric HLA-G from normal trophoblast cells. These results indicate that LILRB1 would mainly bind dimeric HLA-G on the trophoblast *in vivo*, which could represent a mechanism allowing LILRB1^+^ decidual APC to discriminate between the fetal trophoblast and maternal cells expressing conventional HLA-I molecules.

We have shown that soluble HLA-G5 immunoprecipitated from the culture supernatant of transfected cells can also form a β_2_m-associated dimer. However, we were not able to immunoprecipitate any form of soluble HLA-G from culture supernatants of primary trophoblast cells or pre-implantation embryos, consistent with previous reports describing only very low levels of HLA-G5 transcripts 3, 4 and shed HLA-G1 protein *in vivo* 4.

We next investigated the possible function of cell surface HLA-G dimers stimulating LILRB1 on myelomonocytic cells in the decidua. As shown previously, in the presence of HLA-G, MDDC still responded to various activation stimuli to mature to an antigen-presenting phenotype 41. Another study did show a slight reduction in DC maturation markers in the presence of HLA-G tetramers 42, although IL-10 was used during MDDC differentiation in this study and is known to inhibit MDDC maturation 43, 44. In addition to immature DC, antigen presentation by activated DC can also result in suppression of adaptive immune responses, an effect likely to be mediated by concomitant cytokine production 45, 46. Therefore, we measured cytokine production and found that induction of IL-6 and IL-10 was increased by MDDC activated in co-culture with HLA-G^+^ compared to HLA-I null transfectants, whereas inflammatory cytokines such as TNF-α were unaffected. IL-10 has a well characterised role as an immunosuppressive cytokine and has been implicated in the function of regulatory T cells 47. IL-6 is pleiotropic, but has also been shown to have immunosuppressive effects with DC 48. Indeed, non-inflammatory gut mucosal DC co-cultured with intestinal epithelial cells produce IL-6 and IL-10 even in the presence of a pathogen 49. This situation has clear resonance with the local environment in the uterine mucosa where DC are exposed to infiltrating HLA-G^+^ trophoblast cells. These results are also consistent with observations from the myelomonocytic cell line U937, which increased IL-10 and TGF-β1 production after treatment with HLA-G 50.

The most likely functional effect of HLA-G dimers on trophoblast binding to decidual HLA-DR^+^ cells would be down-regulation of damaging maternal T cell responses to allogeneic placental cells. Indeed, in keeping with previous reports, we observed a diminished proliferative response of allogeneic lymphocytes when HLA-G, compared to classical HLA-I molecules, were present on stimulator cells 34–36. Our findings indicate that HLA-G is mediating the inhibitory effect on lymphocyte proliferation by binding to LILRB1 and B2 on responding APC. By removal of HLA-DR^+^ cells from the responder population, we found that HLA-G had no effect on the direct T cell response. This observation is highly relevant to the situation *in vivo* because trophoblast cells do not express allogeneic HLA-A, HLA-B or HLA-II molecules that could be perceived directly by maternal T cells 24. Thus, *in vivo*, T cell recognition of any allogeneic trophoblast- or placenta-specific molecules is likely to be *via* indirect presentation by decidual HLA-DR^+^ cells.

There is increasing evidence for the role of LILRB in inducing tolerogenic DC 42, 51–53, and our study demonstrates how this interaction has particular relevance to the placental bed, the only site where HLA-G dimers are physiologically abundant. There are potential parallels with mechanisms of other immunopriviledged sites, where tissue-specific molecules such as thymic stromal lymphopoietin and vasoactive intestinal peptide modulate DC function 49, 54, 55. We have only studied the functional effect of DC exposed to HLA-G on allogeneic lymphocyte proliferation. However, the altered cytokine production of these DC may also modulate uterine NK cell function 56. HLA-G has now been shown to interact with both NK cells 12 and HLA-DR^+^ cells, together accounting for ∼90% of the leukocytes at the placental implantation site 11. Thus, as placental trophoblast cells infiltrate the uterine mucosa, they can deliver a pregnancy-specific signal to most of the local maternal leukocytes and modify their function to accommodate the feto-placental unit 57.

## Materials and methods

### Cell lines and primary tissue

The cell lines used were: human B lymphoblastoid 721.221 line transfected with HLA-C 58, HLA-G5 28 or HLA-G1 mutated in the leader sequence to abrogate HLA-E expression 14; murine Ltk cells transfected either with human β_2_m alone or with β_2_m and HLA-G together 59; and the choriocarcinoma cell line JEG-3 (ATCC, Rockville, MD). Decidual and placental tissues were obtained from elective terminations of normal first-trimester pregnancies. Ethical approval for the use of these tissues was obtained from the Cambridge Local Research Ethics Committee. Decidual leukocytes and trophoblast cells were isolated as described 25. Briefly, decidual tissue was disaggregated with collagenase and mononuclear cells were purified on Lymphoprep. The trophoblast was released from the chorionic villi by trypsin digestion and macrophages were depleted by adherence to plastic. After culture overnight on fibronectin, 50–80% of trophoblast cells label HLA-G^+^ by flow cytometry with the mAb G233 2.

### HLA-I and LILR antibodies and fusion proteins

Conformationally dependant mAb specific for HLA-G were G233 [2] from our laboratory, 87G 60] from Dr. D. Geraghty, and MEM-G/11 61] from AbCam. BBM.1 (Santa Cruz Biotechnology) specifically recognises β_2_m 62], W6/32 (Serotec) binds molecules encoded by all HLA-I loci 63], Tü149 (from Dr. B. Uchanska-Ziegler) and B1.23.2 (from Dr. P. Le Bouteiller) recognise HLA-B and HLA-C allotypes 23, 64]. MEM-G/1 (Abcam) detects denatured HLA-G 65]. Anti-LILRB1 (M401) and anti-LILRB2 (M422) mAb 66] were supplied by Amgen Inc. Different anti-LILRB1 mAb (HP-F1) 37] and anti-LILRB2 mAb (42D1) 67 were from Dr. M. Lopez-Botet and Dr. M. Colonna, respectively. Isotype control antibodies were from Oxford Biotechnology. A sequenced LILRB1-Fc fusion protein construct 66] from Amgen Inc was transiently transfected into 293T cells using Lipofectamine 2000 (Invitrogen). Fusion protein was purified from supernatant with protein G-Sepharose beads (Amersham Pharmacia Biotech) and assayed by Easy-Titer (Pierce) and reducing SDS-PAGE, where it resolved as a single band at ∼100 kDa.

### Biotinylation, immunoprecipitation, PAGE and Western blotting

Cells were washed with cold PBS, then biotinylated with 0.2 mg/mL EZ-Link Sulfo-NHS-LC-Biotin (Pierce) in pH 8.0 PBS for 30 min at 4°C to prevent endocytic intracellular labelling. Unconjugated reagent was quenched by adding glycine to a concentration of 10 mM. Cells were then lysed in fresh ONYX lysis buffer [20 mM Tris (pH 7.4), 140 mM NaCl, 1 mM EGTA, 1% Triton, 10% glycerol, 50 mM iodoacetamide and protease inhibitor cocktail (Roche)]. This concentration of iodoacetamide has been shown to prevent post-lysis HLA-I multimerisation 30. Labelled lysates were stored at –80°C.

Thawd lysates were incubated for 90 min at 4°C with protein G-Sepharose beads coated with isotype control mAb. Precleared lysates were then immunoprecipitated for 90 min at 4°C with protein G-Sepharose beads precoated with anti-HLA antibodies or LILRB1-Fc. Immunoprecipitates were washed with ONYX containing 3 mM SDS, bound proteins were eluted with NuPAGE LDS Sample Buffer (Invitrogen) and divided into two aliquots; one was denatured by treatment at 95°C for 10 min, the other was denatured and reduced by the same process in the presence of 200 mM 2-mercaptoethanol (Sigma). Denatured samples were stored at –20°C overnight. For immunoprecipitation of soluble HLA-G, transfected 721.221 cell cultures were inoculated at 1 × 10^6^ cells/mL and incubated for 2 days. JEG-3 and primary trophoblast cells were cultured as described for 2 days 2, 25. Human pre-implantation embryo culture supernatants were obtained from Dr. R. Gosden (Cornell University). Supernatants were concentrated by centrifugal filtration if necessary (Amicon, 30 000 MWCO) and a 700-µL aliquot was precleared as before and immunoprecipitated overnight.

Samples were resolved on reducing or non-reducing NuPAGE Bis-Tris 10% gels (Invitrogen), electroblotted and visualised with a streptavidin-HRP conjugate followed by ECL detection (Amersham Biosciences). Unlabelled soluble HLA-G molecules were detected with biotinylated MEM-G/1 followed by streptavidin-HRP. 2D-PAGE was performed by running a non-reducing gel as above, excising the lane and equilibrating for 30 min in reducing buffer (0.125 M Tris pH 8, 3.5 mM SDS, 0.15 M 2-mercaptoethanol). This gel slice was then positioned at 90° along the top of a new gel and run under reducing conditions before Western blotting as above.

### Flow cytometry

Freshly isolated leukocytes were first incubated with human IgG (Sigma) to block Fcγ receptors. Anti-LILRB1 and -LILRB2 antibodies were added and detected with fluorochrome-conjugated secondary antibodies. Free secondary antibody binding sites were blocked with the appropriate species Ig, before staining with directly conjugated CD56, CD3 or HLA-DR mAb (Serotec and Becton Dickinson). Data was acquired with a FACScan flow cytometer and analysed using CellQuest software (Becton Dickinson).

Primary trophoblast cells cultured overnight were removed from the plate with 0.5 mM EDTA. Fcγ receptors were blocked with 40% heat-inactivated human AB serum (Sigma) for G233 mAb staining experiments or with mouse anti-CD32 antibody (Stem Cell Technologies) for LILRB1-Fc staining experiments. Contaminating fetal leukocytes were also labelled with lin-FITC, a cocktail of FITC-conjugated anti-CD3, -CD14, -CD16, -CD19, -CD20 and -CD56 antibodies (Becton Dickinson). Cells were stained with G233 mAb, LILRB1-Fc or IgG1-Fc fragment (Bethyl Laboratories). Primary antibody was detected with PE-conjugated anti-mouse IgG F(ab’)_2_ fragment (Sigma). Fusion protein binding was detected with PE-conjugated anti-human IgG (γ chain specific) secondary antibody (Sigma). In blocking experiments, fusion protein was incubated with anti-LILRB1 or isotype control antibody or cells were incubated with W6/32 or the isotype control antibody prior to staining with fusion protein.

### MDDC experiments

PBMC from healthy donor buffy coats were isolated on Lymphoprep (Axis-Shield, Norway), washed extensively and plated in plastic culture dishes at 2 × 10^7^ cells/mL. After 2 h, non-adherent cells were washed away and the adherent fraction was cultured in RPMI with GM-CSF (100 ng/mL) and IL-4 (40 ng/mL) (Biosource), refreshing the medium as necessary. After 5 days, more than 80% of the cells exhibited a DC-like phenotype (LILRB4^+^, DC-SIGN^+^, CD14^–^) with fewer than 10% of cells CD3^+^. MDDC were stimulated for 12–24 h with 10 ng/mL LPS (Sigma), 500 ng/mL sCD40L (Peprotech) or 20 ng/mL TNF-α, 50 ng/mL IL-6 and 10 ng/mL IL-1β (Biosource, R&D Systems). HLA-I null 721.221 or Ltk parent and HLA-G-transfected cells were irradiated with 1000 rad and added to the MDDC either with the maturation stimulus or throughout their differentiation.

Cells were harvested and stained for flow cytometry with LILRB4-PC5 (Becton Dickinson), PE-conjugated antibodies to CD14 (Becton Dickinson), CD40, CD80, CD83 and CD86 (all Serotec), FITC-conjugated HLA-DR antibody (Becton Dickinson) and unlabelled DC-SIGN (Becton Dickinson), anti-LILRB1 (M401) and -LILRB2 (M422) antibodies followed by PE-conjugated F(ab’)_2_ anti-mouse IgG (Sigma). Medium was analysed for IL-6, IL-8, IL-10 and TNF-α by ELISA (R&D Systems).

### Mixed lymphocyte reaction

Stimulator cells were 721.221 parent (5) or HLA-G1 (105), HLA-G5 and HLA-C (80) transfectants. The HLA-I-transfected cells had similar levels of expression as monitored by BBM.1 staining; the MFI is shown in parentheses above. Stimulator cells were washed, seeded at equal densities and cultured overnight without selection. These cells were then irradiated with 5000 rad and aliquoted into wells of a 96-well plate at varying densities to give stimulator/responder ratios of between 3 : 1 and 0.1 : 1. Responder cells were PBMC from healthy donors, isolated on Lymphoprep and added at 5 × 10^4^ cells/well in a total volume of 300 µL. All donors demonstrated proliferation and when typed retrospectively where shown to be mismatched to the HLA-DR1 epitope expressed by 721.221 cells 58. Donors in the experiments shown in Fig. 7 were DRB1*04/DRB1*07 in panel (A) and DRB1*03/DRB1*13 in panel (B). HLA-DR^+^ cells were removed from responder populations with azide-free HLA-DR-FITC and EasySep FITC-selection kit (Stem Cell Technologies). After 5 days, 1 µCi/well of [^3^H]thymidine was added, cells were harvested after a further 18 h (TomTec 96-well plate harvester), and [^3^H]thymidine incorporation was measured (Wallac Trilux liquid scintillation counter). For blocking experiments, azide-free HP-F1 (3 µL ascites/well), M401, M422 or W6/32 (all 0.05 µg/well) were added immediately. F(ab’)_2_ fragments of W6/32, necessary to avoid antibody-dependant cell-mediated cytotoxicity, were generated by pepsin digestion 68.

### Statistical analysis

For MDDC cytokine production and MLR proliferation experiments, means of replicates of a sample were compared in the presence and absence of HLA-G by two-tailed paired Student's *t*-test for the total number of independent times the experiments were run. Calculations were performed using InStat software, with a *p* value <0.05 regarded as significant.

## Abbreviations:

EVT: extravillous trophoblast
KIR: killer immunoglobulin-like receptor
LILR: leukocyte immunoglobulin-like receptor
MDDC: monocyte-derived dendritic cell
